# The impact of time-updated resting heart rate on cause-specific mortality in a random middle-aged male population: a lifetime follow-up

**DOI:** 10.1007/s00392-020-01714-w

**Published:** 2020-07-21

**Authors:** Xiaotong Cui, Zacharias Mandalenakis, Erik Thunström, Michael Fu, Kurt Svärdsudd, Per-Olof Hansson

**Affiliations:** 1 Department of Cardiology, Shanghai Institute of Cardiovascular Diseases, Zhongshan Hospital, Fudan University, Shanghai, China; 2 Department of Molecular and Clinical Medicine, Institute of Medicine, University of Gothenburg, Sahlgrenska Academy, Gothenburg, Sweden; 3 Department of Medicine, Geriatrics and Emergency Medicine, Sahlgrenska University Hospital/Östra, Diagnosvägen 11, Göteborg, Region Västra Götaland, 416 85 Gothenburg, Sweden; 4 Department of Public Health and Caring Sciences, Family Medicine Section, Uppsala University, Uppsala, Sweden

**Keywords:** Heart rate, Mortality, General population, Cohort study

## Abstract

**Background:**

A high resting heart rate (RHR) is associated with an increase in adverse events. However, the long-term prognostic value in a general population is unclear. We aimed to investigate the impact of RHR, based on both baseline and time-updated values, on mortality in a middle-aged male cohort.

**Methods:**

A random population sample of 852 men, all born in 1913, was followed from age 50 until age 98, with repeated examinations including RHR over a period of 48 years. The impact of baseline and time-updated RHR on cause-specific mortality was assessed using Cox proportional hazard models and cubic spline models.

**Results:**

A baseline RHR of ≥ 90 beats per minute (bpm) was associated with higher all-cause mortality, as compared with an RHR of 60–70 bpm (hazard ratio [HR] 1.60, 95% confidence interval [CI] 1.17–2.19, *P* = 0.003), but not with cardiovascular (CV) mortality. A time-updated RHR of < 60 bpm (HR 1.41, 95% CI 1.07–1.85, *P* = 0.014) and a time-updated RHR of 70–80 bpm (HR 1.34, 95% CI 1.02–1.75, *P* = 0.036) were both associated with higher CV mortality as compared with an RHR of 60–70 bpm after multivariable adjustment. Analyses using cubic spline models confirmed that the association of time-updated RHR with all-cause and CV mortality complied with a U-shaped curve with 60 bpm as a reference.

**Conclusion:**

In this middle-aged male cohort, a time-updated RHR of 60–70 bpm was associated with the lowest CV mortality, suggesting that a time-updated RHR could be a useful long-term prognostic index in the general population.

**Graphic abstract:**

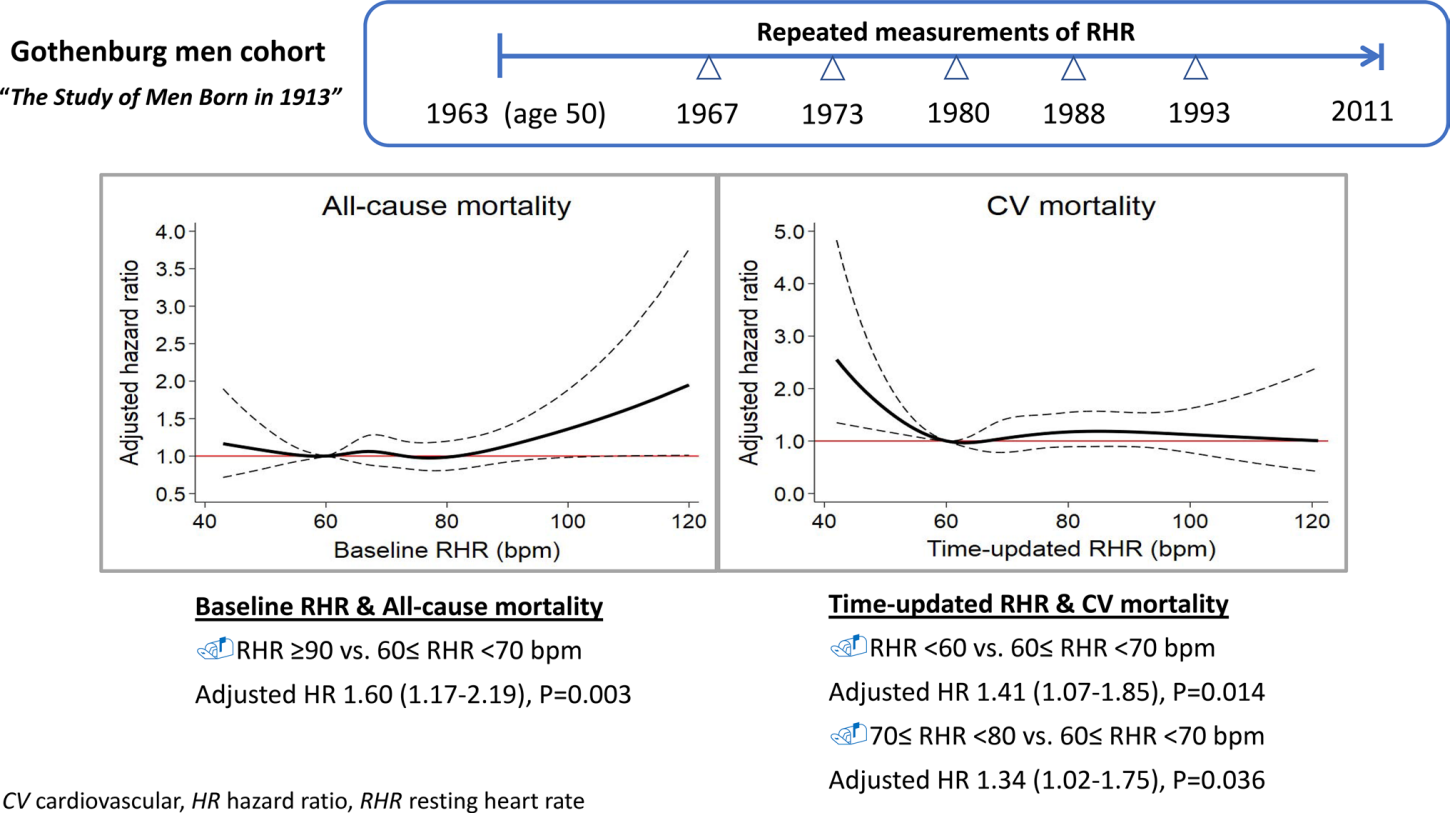

## Introduction

An elevated resting heart rate (RHR) is related to both increased cardiovascular (CV) morbidity and mortality in the general population [1–10]. Previous studies have shown that an increase in RHR of 10 beats per minute (bpm) will increase the risk of incident all-cause mortality by 17% [9]. Moreover, the change in RHR over a period of several years is also associated with mortality or CV events [10–14]. However, the RHR varies throughout life and there is still a suspended question of whether a time-updated RHR is also of prognostic value in relation to mortality. It has recently been demonstrated that an increase in time-updated RHR is associated with a higher risk of mortality in patients with heart failure or with chronic obstructive pulmonary disease (COPD) [15–17]. However, whether this relationship exists in the general population remains unknown. ‘The Study of Men Born in 1913’ is a random prospective cohort study from the general male population, which has been re-investigated several times during a 48-year follow-up. The present study aimed to investigate the impact of both baseline and time-updated RHR on the incidence of cause-specific mortality in this general population sample.

## Methods

### Study population

‘The Study of Men Born in 1913’ is a prospective cohort established in 1963 and comprises one-third of the male population born in 1913 and living in Gothenburg, Sweden, at the time of sampling. Details of the cohort and the sampling process have been given previously [18–21]. At baseline in 1963, 855 men (87.9% of the whole sample who fulfilled the inclusion criteria) agreed to participate in the study. Informed consent was obtained at each examination, orally during the first examinations, as required at the time, and in writing later on, according to the Helsinki Declaration. Research ethics approval was obtained on several occasions, first from the Research Ethics Committees in Gothenburg and Uppsala, Sweden, and later from the Regional Ethics Review Board, Uppsala, Sweden, No. 2011/304. The only exclusion criterion in the present study was a history of atrial fibrillation before the baseline examination.

### Assessments and data collection

**Fig. 1:**
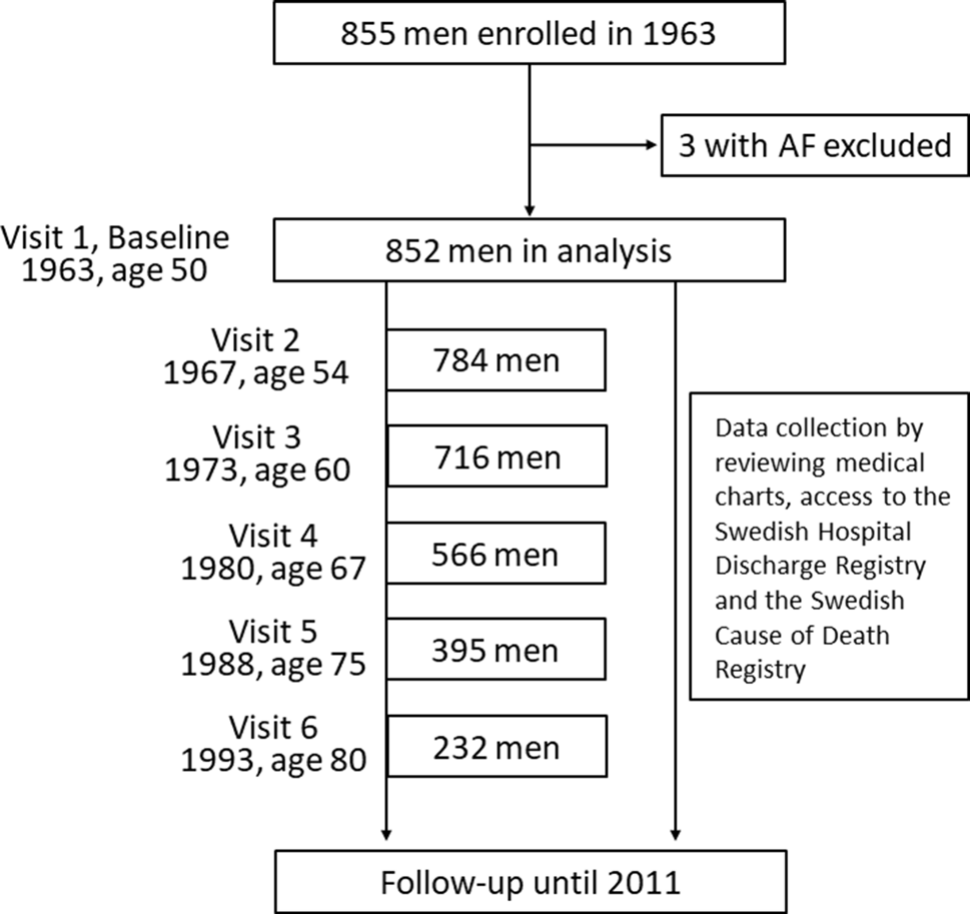
Flowchart of the study. *AF* atrial fibrillation

The participants were examined at baseline in 1963 when they were all 50 years old and re-examined in 1967 (at age 54 years), 1973 (age 60), 1980 (age 67), 1988 (age 75), and 1993 (age 80) (Fig. 1). Clinical examinations including a 12-lead electrocardiogram (ECG) were performed on each visit. Medical histories were collected by questionnaires.

Weight and height were measured, and body mass index (BMI) was calculated as weight (kg)/height (m)^2^. Waist circumference was measured at the level of the umbilicus. Blood pressure was measured using a standard mercury sphygmomanometer. Hypertension was defined as a confirmed medical history, or the use of antihypertensive medications, or current systolic blood pressure (SBP) of ≥ 140 mmHg or diastolic blood pressure (DBP) of ≥ 90 mmHg.

Atrial fibrillation was defined as atrial fibrillation or atrial flutter detected on the ECG recording at any of the screening examinations or by diagnoses from the Swedish Hospital Discharge Register. Smoking habits were asked about in the questionnaire and classified as never smokers, former smokers, and current smokers. In the Cox analysis, smoking was treated as a binary variable (current smoker or not).

Physical activity during leisure time was assessed with the four-grade Saltin Grimby Physical Activity Scale and graded as 1, sedentary; 2, some light physical activity during leisure time, such as walking or light gardening for at least 4 h a week; 3, regular moderate physical activity for at least 3 h a week; and 4, regular intense physical training for competitive sports [22]. Serum total cholesterol and triglyceride were analysed from venous blood samples drawn after an overnight fast. Diabetes was defined as a diagnosis of diabetes in the medical history.

### Measurements of RHR and definition of time-updated RHR

The RHR was recorded at rest using a standard 12-lead ECG with paper speed 50 mm/s and voltage calibration 10 mm/mV at the baseline examination in 1963 and subsequent re-examinations in 1967, 1973, 1980, 1988, and 1993. The ECGs were evaluated by physicians who were blinded to the other clinical information relating to the participants at the time. The time-updated RHR was defined as the most recent available RHR for each man before the endpoint occurred. This means that, when analysing the association between the time-updated RHR and the outcome, a participant’s ‘baseline’ RHR was carried forward until the next follow-up visit, at which time the new RHR obtained on this visit was adopted and subsequently carried forward again until the next visit. The RHR could, therefore, be updated up to five times after the baseline examination. To avoid the influence of atrial fibrillation, the RHR was only updated until the last visit before the atrial fibrillation diagnosis.

### Follow-up and endpoint identification

All the participants were followed up prospectively until death or until 31 December 2011 for survivors. Endpoints in the study included all-cause mortality, CV mortality, and non-CV mortality. CV mortality was defined as deaths caused by any cardiac disease (ICD-9 codes 393-398, 410-414, 420-429, ICD-10 codes I00-I09, I11, I13, and I20-51), primary hypertension (401-405, I10), cerebrovascular diseases (430-438, I60-I69), and peripheral arterial diseases (440.2-440.4, 444.2, I70.2-I70.7, I74.2-I74.4). The follow-up and outcome data were collected by questionnaires on each visit, reviews of medical charts, and access to the Swedish Hospital Discharge Registry and the Swedish Cause of Death Registry.

### Statistical analysis

Baseline characteristics were shown by dividing the participants into five groups according to RHR at baseline (RHR < 60, 60 to  < 70, 70 to  < 80, 80 to  < 90, and ≥ 90 bpm). Continuous variables were presented as the mean ± standard deviation or median (interquartile ranges) and categorical variables were given as counts and percentages. For comparisons between groups, the ‘nptrend’ test was used for continuous variables and unordered categorical variables, while Spearman’s rank correlation test was used for two-way ordered categorical variables.

The impact of baseline and time-updated RHR on all-cause, CV, and HF mortality was assessed by univariable and multivariable Cox proportional hazard models, in which the RHR was entered as a continuous variable, evaluated by each 10 bpm increase and as a categorical variable stratified in the same way as in Table 1. The stratification of 60–70 bpm was treated as a reference level when the RHR was studied as a categorical variable. For the baseline RHR, confounders adjusted in the multivariable Cox models included SBP, BMI, smoking status, diabetes, and total cholesterol collected at baseline. For the time-updated RHR, confounders adjusted in the multivariable Cox models included time-updated SBP, time-updated BMI, time-updated smoking status, time-updated diabetes, and time-updated total cholesterol. Furthermore, we investigated the association of baseline and time-updated RHR with cause-specific mortality by utilising an adjusted Cox model using a restricted cubic spline with five knots and a reference value of 60 bpm. Confounders adjusted in the cubic model were consistent with those shown above in the multivariable Cox models.

**Table 1:** Baseline characteristics by RHR examined at 1963

Variables	Overall *n* = 852	RHR < 60 *n* = 196	60 ≤ RHR < 70 *n* = 298	70 ≤ RHR < 80 *n* = 224	80 ≤ RHR < 90 *n* = 83	RHR ≥ 90 *n* = 51	*P* value
Mean RHR, bpm	69 ± 12	55 ± 4	64 ± 3	74 ± 3	84 ± 3	97 ± 7	
Median RHR, bpm	67 (60–75)	56 (52–58)	65 (62–67)	73 (71–76)	83 (81–87)	97 (91–102)	
Time-updated RHR, bpm	68 (61–79)	61 (54–68)	66 (60–75)	72 (66–80)	79 (69–90)	85 (72–98)	
SBP, mmHg	138.3 ± 20.9	133.9 ± 18.9	135.9 ± 20.3	140.4 ± 21.2	148.3 ± 22.7	143.2 ± 21.2	< 0.001
DBP, mmHg	91.6 ± 13.2	87.2 ± 11.7	90.0 ± 13.0	94.1 ± 13.7	97.1 ± 12.5	97.4 ± 12.0	< 0.001
BMI, kg/m^2^	24.8 ± 3.2	24.8 ± 3.3	24.8 ± 2.9	24.9 ± 3.3	24.6 ± 2.5	24.4 ± 4.1	0.492
Smoking							
Never smoker	207 (24.3)	47 (24.0)	69 (23.1)	59 (26.3)	19 (22.9)	13 (25.5)	0.986
Former smoker	167 (19.6)	43 (21.9)	59 (19.8)	37 (16.5)	17 (20.5)	11 (21.6)	
Current smoker	478 (56.1)	106 (54.1)	170 (57.1)	128 (57.2)	47 (56.6)	27 (52.9)	
Leisure physical activity							
Sedentary	294 (35.3)	72 (37.3)	91 (31.3)	80 (35.7)	30 (38.0)	21 (45.7)	0.542
Moderate activity	268 (32.2)	54 (28.0)	110 (37.8)	71 (31.7)	22 (27.8)	11 (23.9)	
Regular exercise or intense training	271 (32.5)	67 (34.7)	90 (30.9)	73 (32.6)	27 (34.2)	14 (30.4)	
Hypertension	577 (67.7)	108 (55.1)	189 (63.4)	171 (76.3)	69 (83.1)	40 (78.4)	< 0.001
Diabetes	6 (0.7)	0	0	1 (0.5)	2 (2.4)	3 (5.9)	< 0.001
Total s-cholesterol, mmol/L	6.4 ± 1.1	6.3 ± 1.0	6.4 ± 1.1	6.5 ± 1.1	6.6 ± 1.3	6.6 ± 1.3	0.037
s-Triglycerides, mmol/L	1.3 ± 0.8	1.1 ± 0.5	1.2 ± 0.7	1.3 ± 1.1	1.3 ± 1.0	1.4 ± 0.7	< 0.001

Values are given as n (%), mean ± standard deviation or median (interquartile ranges). *BMI* body mass index, *DBP* diastolic blood pressure, *RHR* resting heart rate, *SBP* systolic blood pressure

Statistical significance was set at *P* < 0.05 (two-tailed). All the statistical analyses were performed with Stata 16.0 (Stata Corp LLC, College Station, Texas, USA).

## Results

Three of 855 participants were diagnosed with atrial fibrillation before the baseline examination and were excluded, leaving 852 men in the current analysis. The characteristics of the study population are shown in Table 1. At baseline, men with a higher RHR had higher levels of SBP, DBP, serum total cholesterol, and triglycerides, and more frequently had hypertension and diabetes.

During the study period, 829 men died (mortality rate 34.3/1000 person-years), of whom 412 men died from CV diseases (CV mortality rate 17.0/1000 person-years) and 417 men died from non-CV causes (non-CV mortality rate 17.2/1000 person-years), Table 2.

**Table 2:** Impact of baseline and time-updated RHR on cause-specific mortality

	*n*	All-cause mortality			CV mortality			Non-CV mortality		
		Events and event rate*	HR (95% CI) *P* value		Events and event rate*	HR (95% CI) *P* value		Events and event rate*	HR (95% CI) P value	
			Unadjusted	Adjusted**		Unadjusted	Adjusted**		Unadjusted	Adjusted**
Baseline RHR, per 10 bpm increase	852	829 (34.3)	1.06 (1.00–1.12) 0.060	1.02 (0.96–1.09) 0.451	412 (17.0)	1.02 (0.94–1.11) 0.647	0.95 (0.87–1.04) 0.273	417 (17.2)	1.10 (1.01–1.19) 0.028	1.10 (1.01–1.20) 0.024
Baseline RHR										
RHR < 60	196	190 (33.4)	1.06 (0.88–1.27) 0.561	1.05 (0.88–1.26) 0.579	97 (17.0)	1.03 (0.80–1.33) 0.810	1.03 (0.80–1.33) 0.813	93 (16.3)	1.08 (0.83–1.41) 0.557	1.08 (0.83–1.40) 0.582
60 ≤ RHR < 70	298	290 (32.9)	1[Reference]	1[Reference]	152 (17.3)	1[Reference]	1[Reference]	138 (15.7)	1[Reference]	1[Reference]
70 ≤ RHR < 80	224	218 (35.1)	1.10 (0.92–1.31) 0.294	1.05 (0.88–1.25) 0.604	99 (16.0)	0.95 (0.74–1.23) 0.703	0.90 (0.70–1.16) 0.411	119 (19.2)	1.26 (0.99–1.61) 0.065	1.22 (0.95–1.57) 0.111
80 ≤ RHR < 90	83	81 (35.4)	1.08 (0.84–1.38) 0.554	0.93 (0.72–1.22) 0.614	44 (19.3)	1.12 (0.80–1.56) 0.523	0.88 (0.62–1.26) 0.499	37 (16.2)	1.04 (0.72–1.49) 0.852	1.02 (0.69–1.50) 0.934
RHR ≥ 90	51	50 (41.7)	1.59 (1.18–2.16) 0.002	1.60 (1.17–2.19) 0.003	20 (16.7)	1.22 (0.76–1.94) 0.409	1.07 (0.63–1.79) 0.806	30 (25.0)	2.01 (1.35–2.99) 0.001	2.19 (1.47–2.38) < 0.001
Time-updated RHR, per 10 bpm increase	852	829	1.03 (0.98–1.08) 0.235	1.02 (0.97–1.07) 0.466	412	1.00 (0.93–1.07) 0.973	0.98 (0.91–1.05) 0.528	417	1.06 (0.99–1.14) 0.088	1.06 (0.99–1.14) 0.084
Time-updated RHR										
RHR < 60	186	180	1.08 (0.89–1.31) 0.430	1.10 (0.91–1.33) 0.339	103	1.43 (1.09–1.87) 0.009	1.41 (1.07–1.85) 0.014	77	0.81 (0.61–1.07) 0.140	0.86 (0.65–1.14) 0.282
60 ≤ RHR < 70	269	262	1[Reference]	1[Reference]	114	1[Reference]	1[Reference]	148	1[Reference]	1[Reference]
70 ≤ RHR < 80	196	191	1.12 (0.93–1.35) 0.230	1.11 (0.92–1.34) 0.267	103	1.40 (1.07–1.83) 0.013	1.34 (1.02–1.75) 0.036	88	0.91 (0.70–1.18) 0.468	0.94 (0.72–1.23) 0.660
80 ≤ RHR < 90	108	105	1.14 (0.91–1.43) 0.255	1.07 (0.85–1.35) 0.565	49	1.21 (0.86–1.69) 0.274	1.04 (0.73–1.47) 0.835	56	1.09 (0.80–1.48) 0.584	1.10 (0.80–1.50) 0.571
RHR ≥ 90	93	91	1.23 (0.97–1.57) 0.090	1.22 (0.95–1.56) 0.115	43	1.35 (0.95–1.92) 0.098	1.26 (0.88–1.81) 0.213	48	1.14 (0.82–1.59) 0.427	1.21 (0.86–1.68) 0.273

*CI* confidence interval, *CV* cardiovascular, *HR* hazard ratio, *RHR* resting heart rate

*Event rate per 1,000 person-years, only calculated by baseline RHR

**For baseline RHR, adjusted for SBP, BMI, smoking status, diabetes, and total cholesterol. For time-updated RHR, adjusted for time-updated SBP, time-updated BMI, time-updated smoking status, time-updated diabetes, and time-updated total cholesterol

### Baseline RHR and cause-specific mortality

Used as a continuous variable, the adjusted hazard ratio (HR) for each 10 bpm increase in baseline RHR in relation to all-cause mortality was 1.02, 95% confidence interval (CI) 0.96–1.09, *P* = 0.451, Table 2. The all-cause mortality incidence in those with an RHR of ≥ 90 bpm increased by 60% (HR 1.60, 95% CI 1.17–2.19, *P* = 0.003) as compared with the group of men with an RHR of 60–70 bpm.

**Fig. 2:**
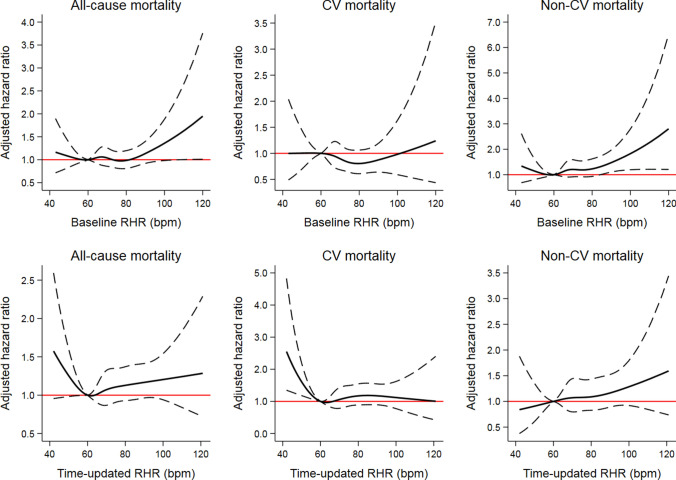
Association of baseline and time-updated RHR with cause-specific mortality by adjusted cubic spline model. The black dashed curves represent the upper and lower 95% confidence limits, respectively. The horizontal red line represents the adjusted hazard ratio of 1. *CV* cardiovascular, *RHR* resting heart rate

Further analyses showed that a high baseline RHR was a statistically significant predictor of non-CV mortality with HR 1.10 (95% CI 1.01–1.20, *P* = 0.024) per 10 bpm increase and HR 2.19 (95% CI 1.47–2.38, *P* < 0.001) for an RHR of ≥ 90 bpm versus 60–70 bpm, while no significant association was detected between baseline RHR and CV mortality. In analyses using the restricted cubic spline model, an RHR at baseline above 105 bpm was related to a higher risk of all-cause mortality and an RHR at baseline above 85 bpm was related to a higher risk of non-CV mortality compared with an RHR of 60 bpm, Fig. 2. The baseline RHR was not correlated to CV mortality in the adjusted Cox analysis using the cubic spline model.

### Time-updated RHR and cause-specific mortality

The time-updated RHR was not significantly associated with all-cause mortality or non-CV mortality, either as a continuous variable or as a categorical variable, despite a trend similar to the baseline RHR, Table 2. However, a time-updated RHR of < 60 bpm (HR 1.41, 95% CI 1.07–1.85, *P* = 0.014) and a time-updated RHR of 70–80 bpm (HR 1.34, 95% CI 1.02–1.75, *P* = 0.036) were both associated with a higher risk of CV mortality as compared with the time-updated RHR of 60–70 bpm in the multivariable model.

The Cox analyses with the cubic spline model revealed a U-shaped trend for the time-updated RHR of < 60 bpm and > 60 bpm, both of which showed an incidence increase in all-cause mortality and CV mortality, Fig. 2. Non-CV mortality showed a one-way rising curve across the whole time-updated RHR spectrum, although no significance was achieved.

## Discussion

In a 48-year prospective follow-up of a random general population sample of 50-year-old men, we found that a higher baseline RHR was associated with a higher risk of all-cause and non-CV mortality but not CV mortality. However, the time-updated RHR with 60 bpm as a reference showed a U-shaped curve and was closely associated with CV mortality. Our findings suggest that, in addition to an RHR in middle age, a time-updated recent RHR may also be a useful prognostic index in the general population.

The established relationship between the baseline RHR and the risk of mortality in the general population has been confirmed by two recent meta-analyses comprising 46 and 87 studies, respectively [6, 9]. However, people’s heart rate may not remain stable throughout their lives. Therefore, whether or not a time-updated heart rate has prognostic importance should also be an interesting and critical question. The research team of the CHARM (candesartan in heart failure: assessment of reduction in mortality and morbidity) and TOPCAT (Treatment of Preserved Cardiac Function Heart Failure With an Aldosterone Antagonist) studies demonstrated in both cohorts that a time-updated RHR of > 76 bpm was associated with a steep rise in the risk of CV mortality or heart failure hospitalisation in chronic heart failure patients [15, 16]. Omlor et al. found that the risk of all-cause mortality in COPD patients increased by 79% in those with a time-updated RHR of > 72 bpm compared with an RHR of ≤ 72 bpm [17].

To our knowledge, the present study is the first to investigate the association between a time-updated RHR and prognosis in the general population. Our analyses indicate that a time-updated RHR is independently associated with CV mortality in the studied male cohort, providing additional evidence that an RHR recorded at any time is also of prognostic importance and should receive attention not only in patients with CV or pulmonary diseases but also in the general population.

Our analyses demonstrated a U-shaped impact of time-updated RHR on CV mortality, with elevated mortality when the RHR diverged (either lower or higher) from 60 to 70 bpm. These findings differ slightly from those in the previous studies with regard to the effect of a time-updated RHR of < 60 bpm [15–17]. This discrepancy is probably due to different study populations. In a general population sample, only a subgroup may have cardiovascular or pulmonary diseases. Our results and previous findings together emphasise that a relatively low RHR is a determinant of a better long-term prognosis both among patients with established cardiovascular diseases and in the general male population.

This finding is of importance, as the impact of a time-updated RHR on the long-term outcome in a general population has not previously been studied during a follow-up period of almost 5 decades, making our data unique. Moreover, the present study has extended our knowledge by showing that both an extremely low and high time-updated RHR are associated with an adverse CV outcome, indicating that an RHR at the lower limit of normal heart rate range is desirable and should, therefore, be achieved as a preventive goal even in a general population.

The pathophysiological explanations of our findings are probably multifactorial. A low time-updated RHR may represent some recently occurred underlying pathophysiological changes ranging from conduction system disturbances to other potential arrhythmias [23]. A reduced heart rate could result in the dispersion of atrial repolarisation, which would, in turn, initiate CV events [24, 25]. For instance, a low heart rate has been shown to be a predictor of incident atrial fibrillation in healthy middle-aged men [26]. Additionally, bradycardia may also reduce the cardiac output and the peripheral perfusion in certain pathological situations, or even reduce the ability to respond to stressful events. Although a high RHR has been shown to exert harmful effects on the progression of coronary atherosclerosis, the occurrence of myocardial ischaemia, the incidence of atrial and ventricular arrhythmias, and the attenuation of left ventricular function [27], the present study indicates that the risk may only increase continuously at heart rates above 60–70 bpm. Instead, a low heart rate appears not to be beneficial and indicates increased mortality.

Although several previous studies have found a stronger association with mortality for time-updated RHR than for baseline RHR [15–17], our study found that baseline RHR better predicts all-cause mortality. The main reason for this different finding is probably differences in study populations. While most previous studies were on COPD or HF patients, we studied a general population sample with a low rate of cardiovascular disease at baseline.

### Strengths and limitations

The strengths of the present study include the fact that we investigated a randomly sampled cohort of the same age and sex from the general population, followed prospectively for 48 years with comprehensive identifications of outcome events by accessing the Swedish National Health Registry and the Swedish Cause of Death Registry. The RHRs were repeatedly examined up to six times between 1963 and 1993, allowing us to assess the impact of a time-updated RHR on mortality. Some limitations should also be recognised.

One limitation is that we only included male participants in the present study and conclusions about RHR in middle-aged women may differ. Nevertheless, according to the study by Omlor et al. in COPD patients, there was no evidence supporting significant sex-related differences in relation to the effect of time-updated RHR on mortality, although they found that male sex was associated with a higher time-updated RHR [17]. Another limitation is the fairly small sample size, which may limit the power of the test to some extent. However, our cohort had a very long-term follow-up and this would make up for some shortcomings caused by the relatively small sample size.

## Conclusions

During a 48-year follow-up of this random middle-aged male cohort from the general population, a time-updated RHR of 60–70 bpm was associated with the lowest CV mortality, suggesting that the time-updated RHR could be a useful prognostic index in the long run in the general population and supporting the importance of measuring RHR intermittently throughout life.

## Data Availability

Data could be available under reasonable request after authors’ evaluation and agreement.
